# Supplementary material for: Chewing Behavior Attenuates Lung-Metastasis-Promoting Effects of Chronic Stress in Breast-Cancer Lung-Metastasis Model Mice
Source: Cancers (Basel). 2022 Dec 1;14(23):5950. doi: 10.3390/cancers14235950 (PMC9740082; doi:10.3390/cancers14235950)
Supplement: Supplementary file 1 [file cancers-14-05950-s001.zip › cancers-1997202-supplementary/File S1.pdf]

Figure S1. The uncropped immunoblotting images

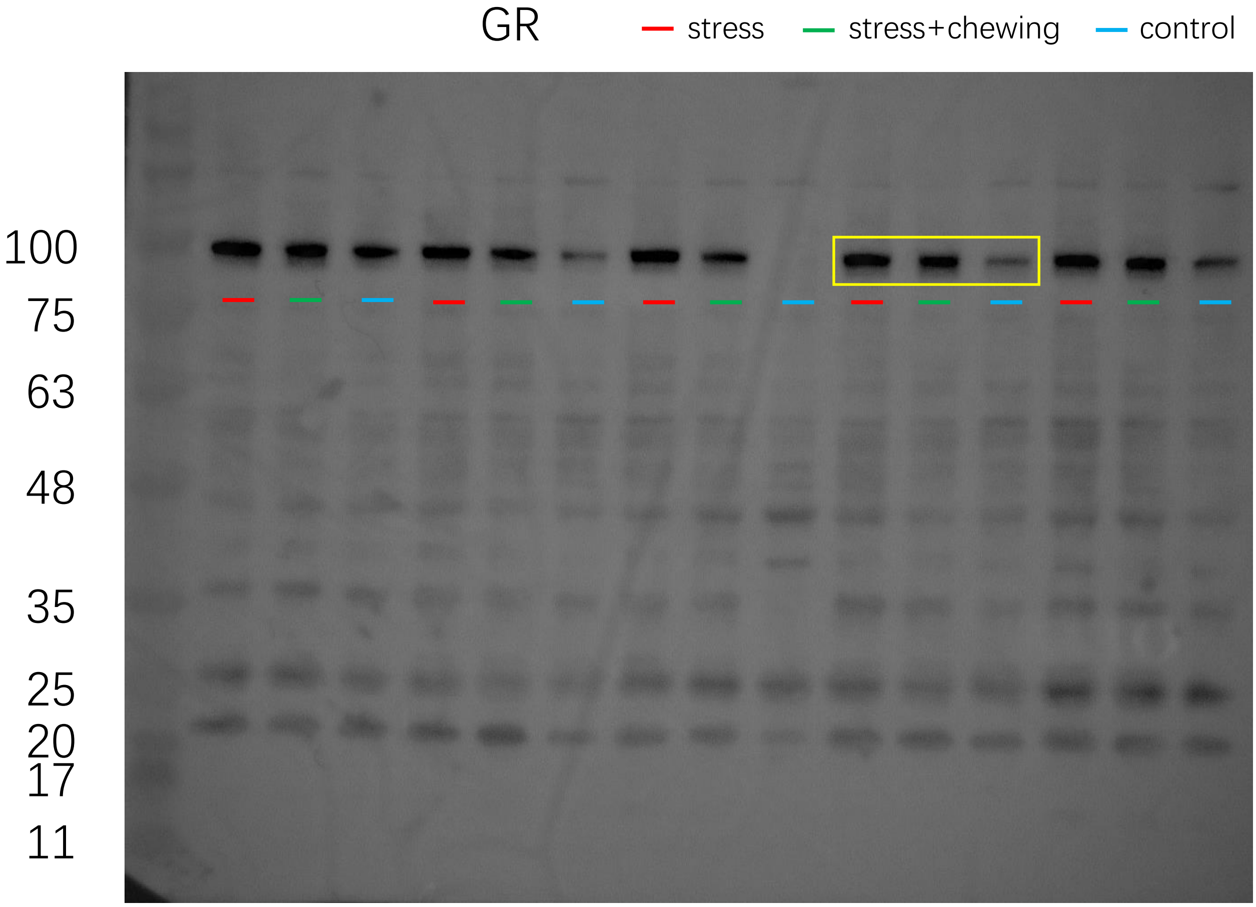

Fig.2E

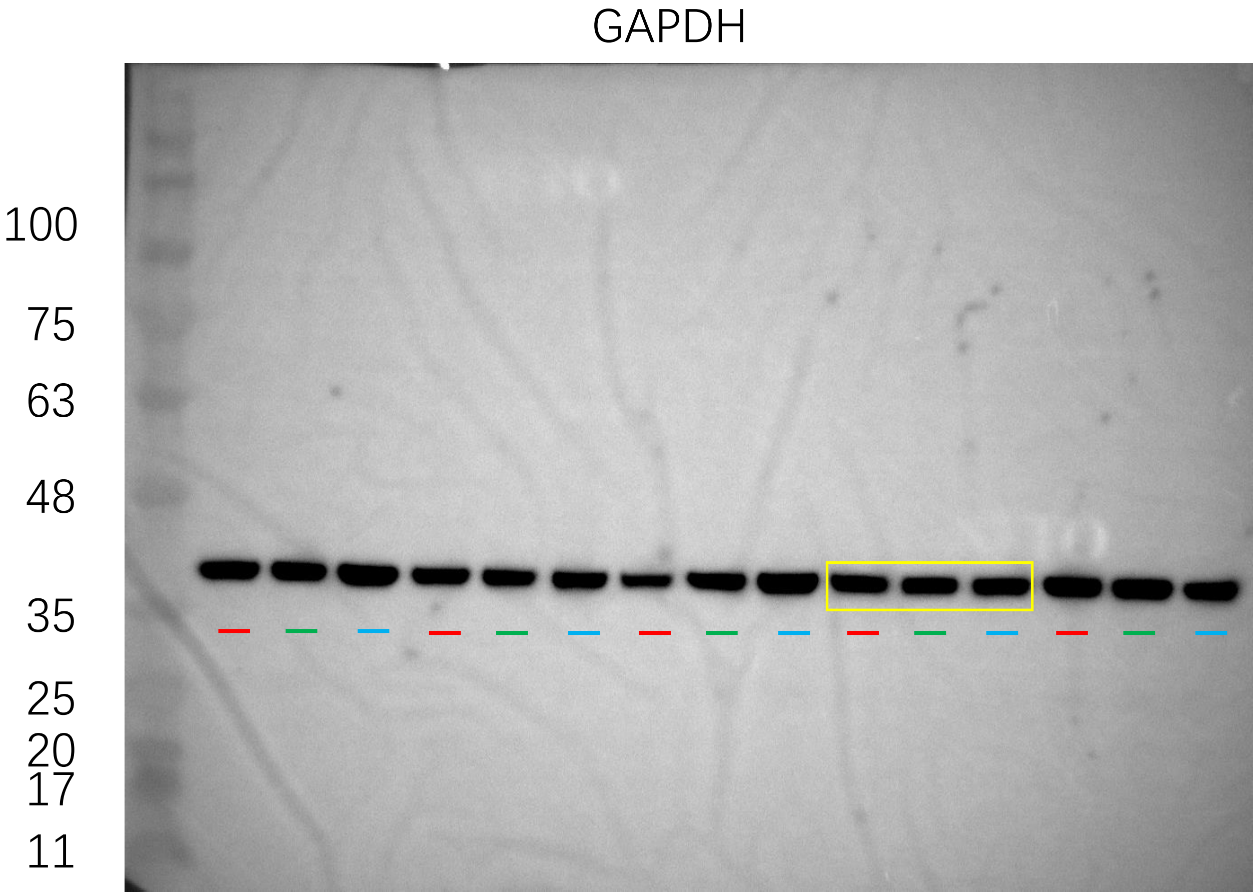

Fig.2E

β2AR

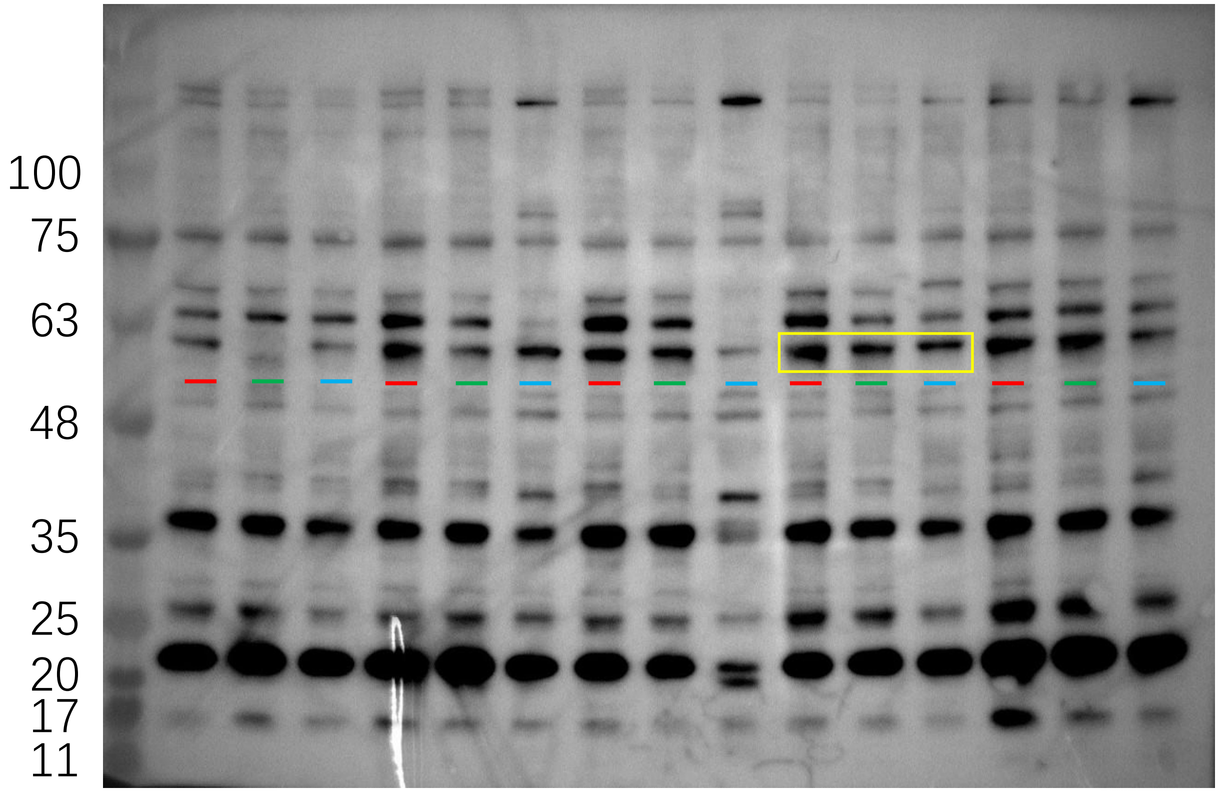

Fig.2F

GAPDH

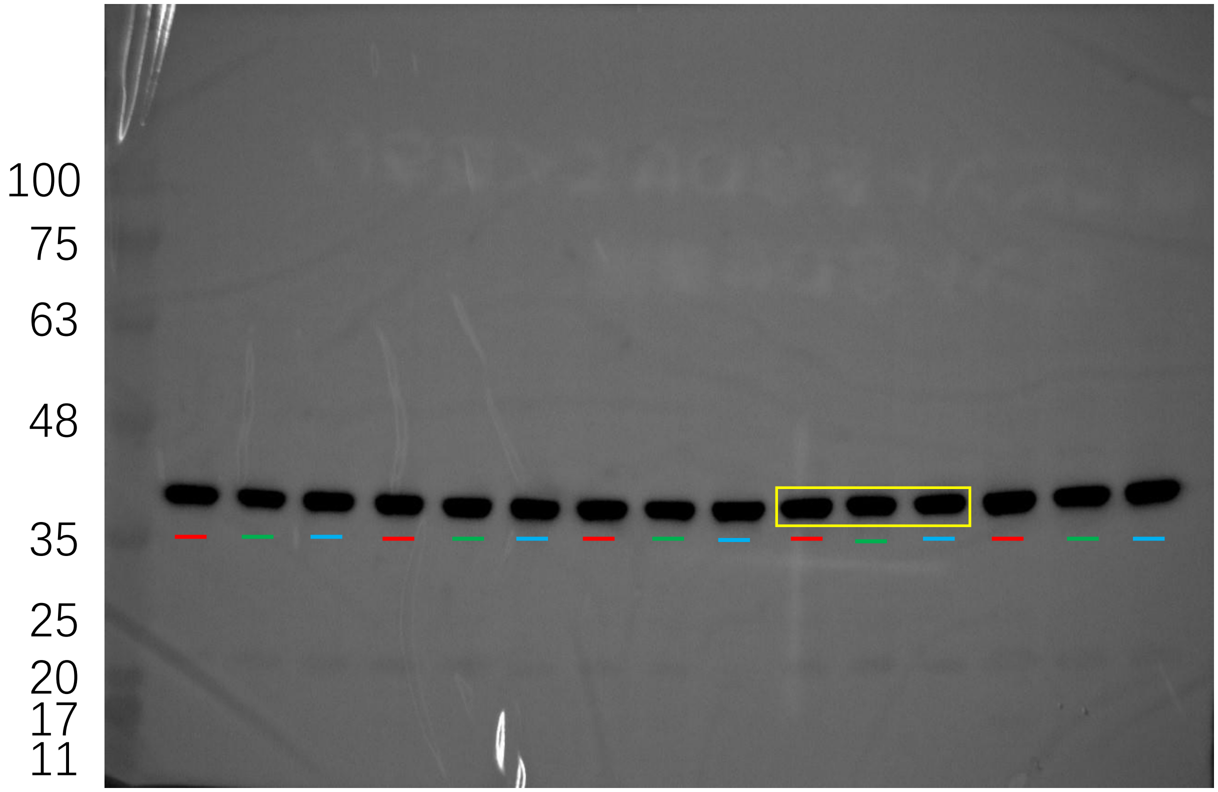

Fig.2F

TNF-α

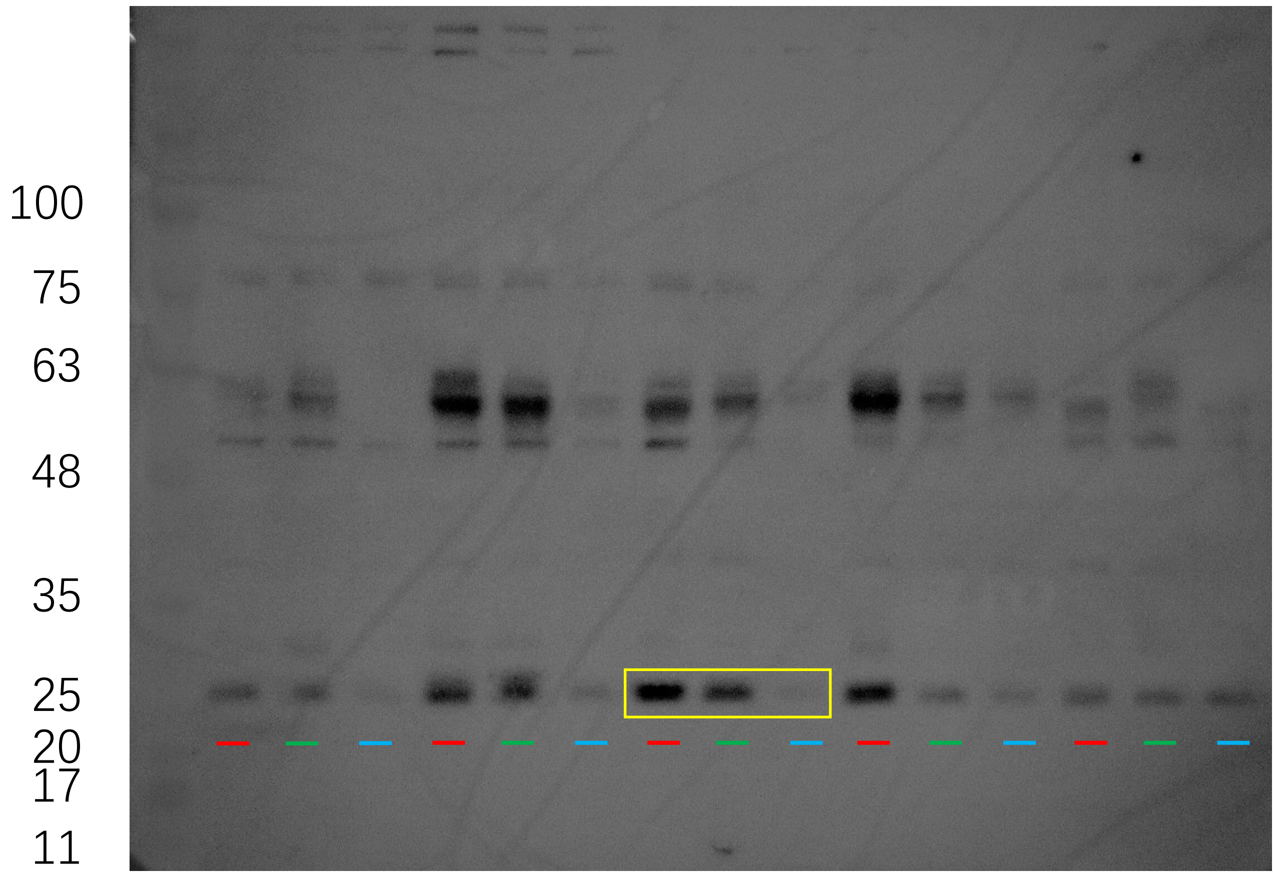

Fig.3A

GAPDH

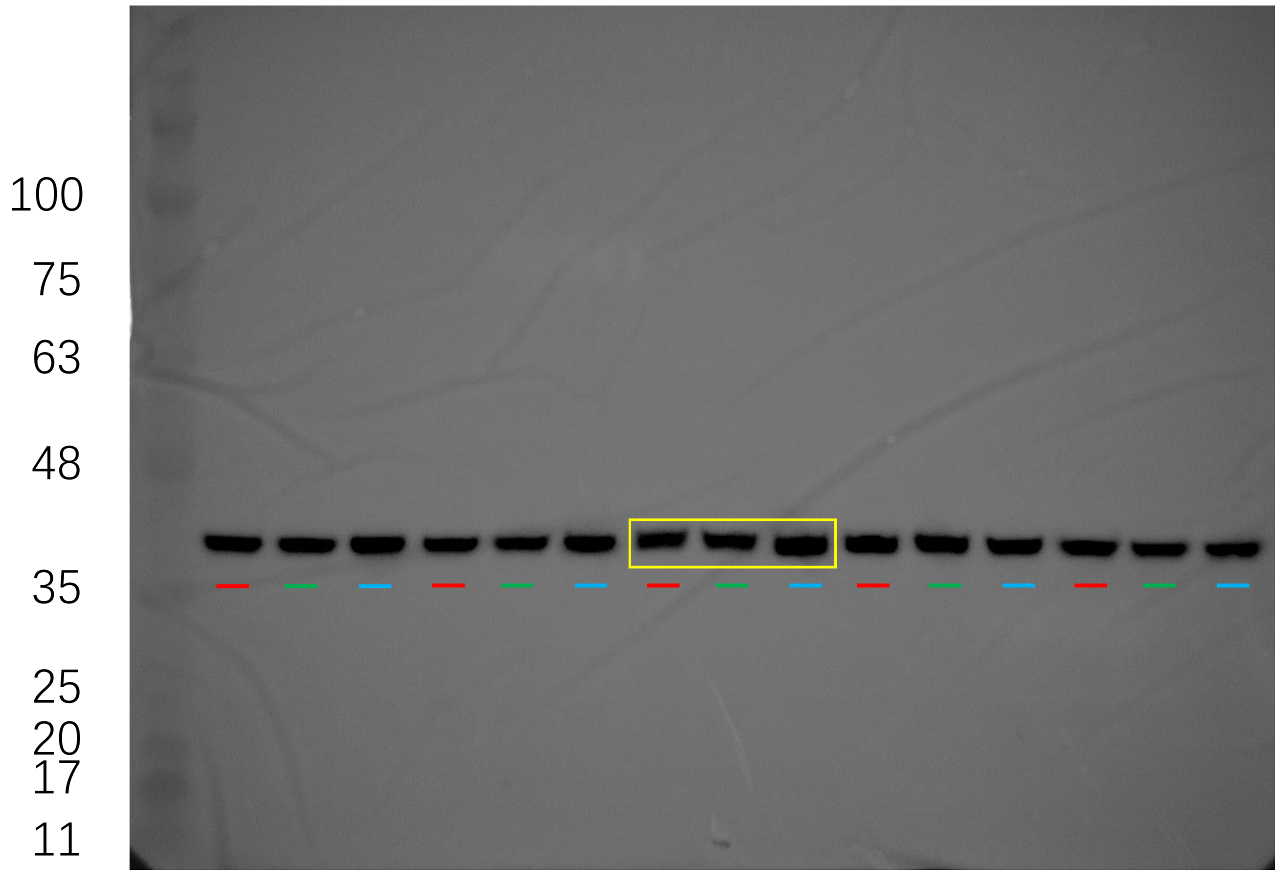

Fig.3A

TGF-β

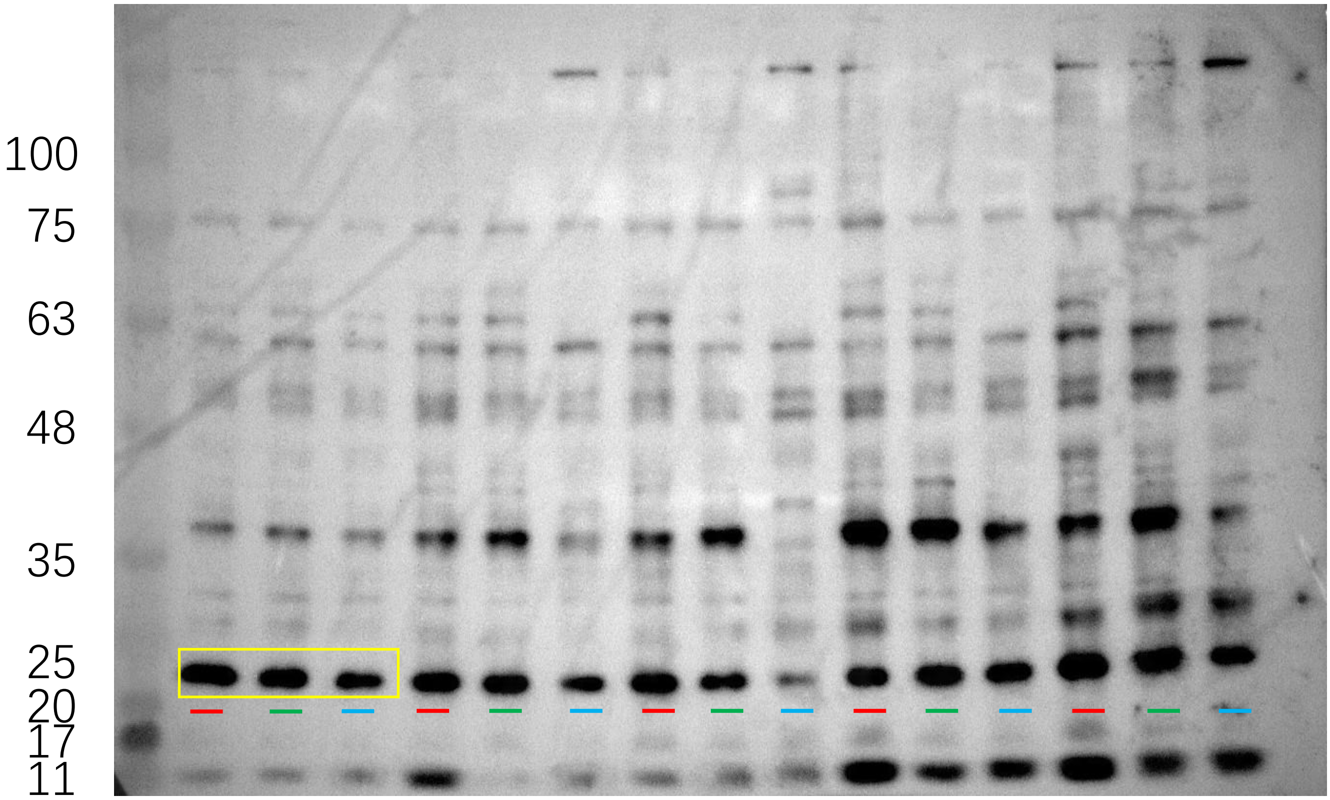

Fig.3B

GAPDH

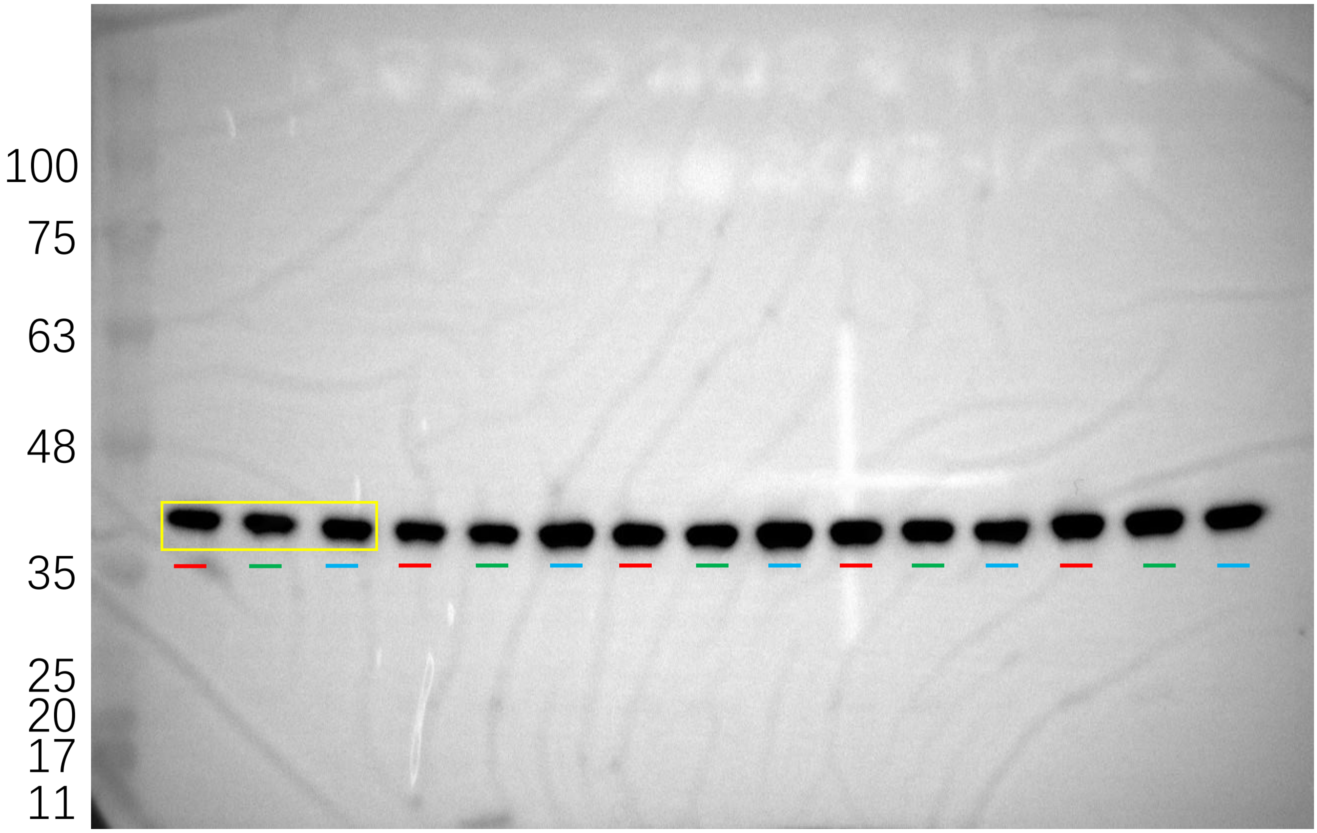

Fig.3B

VEGF

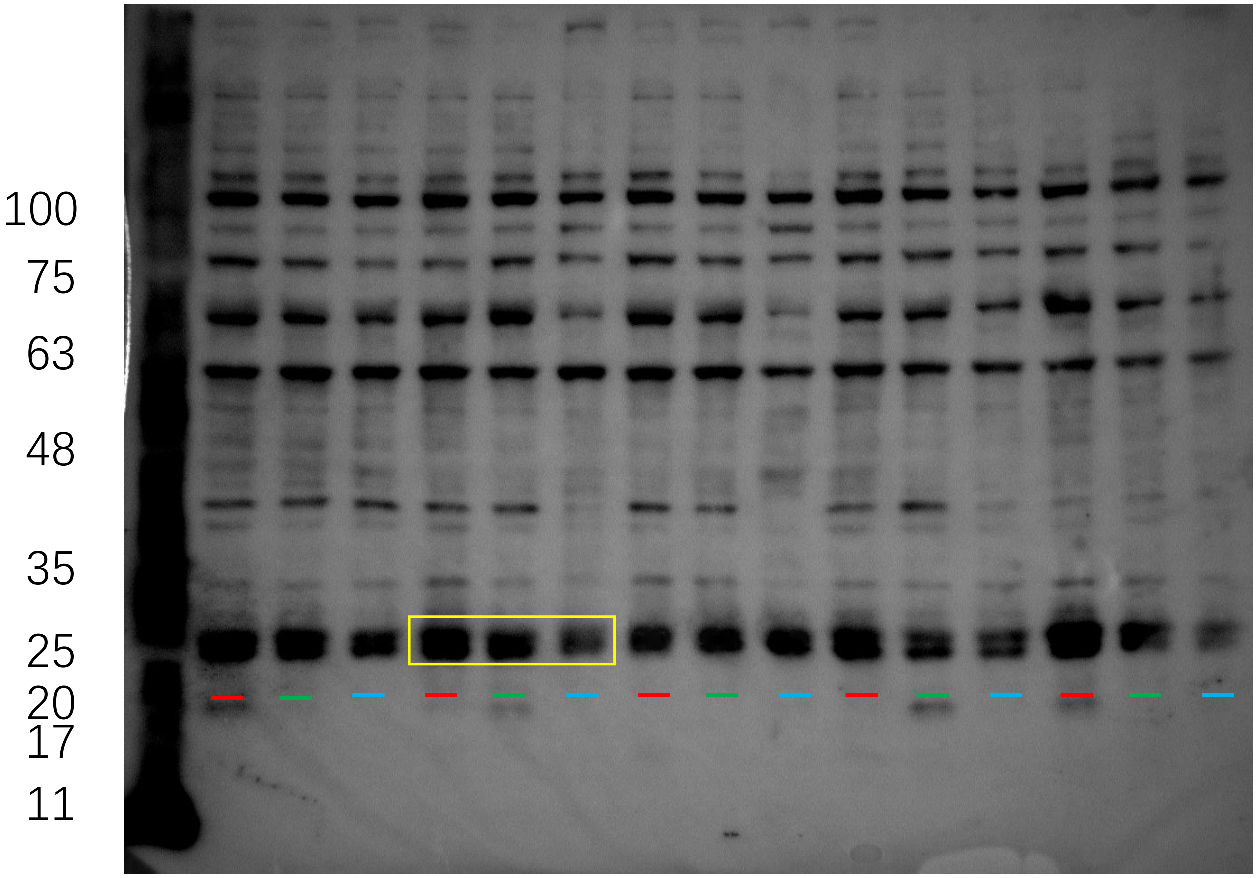

Fig.3C

GAPDH

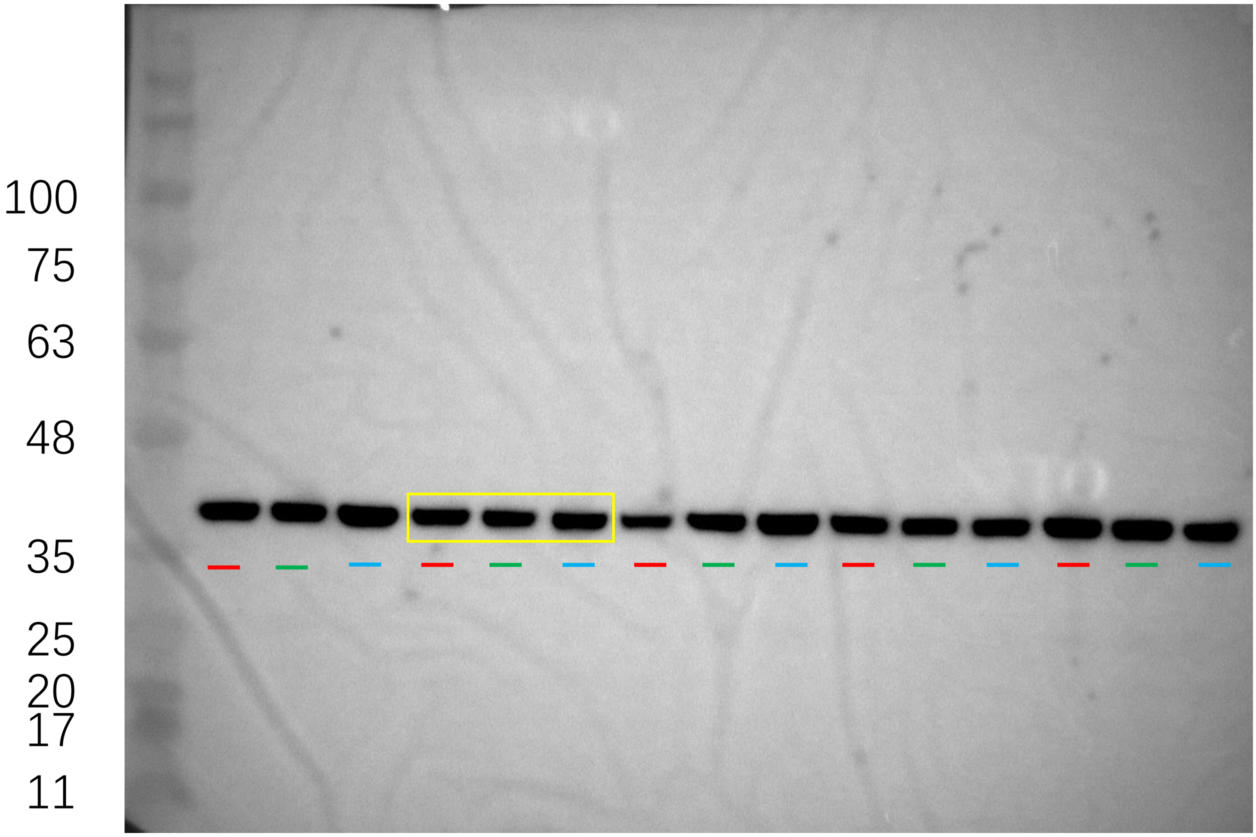

Fig.3C

MMP2

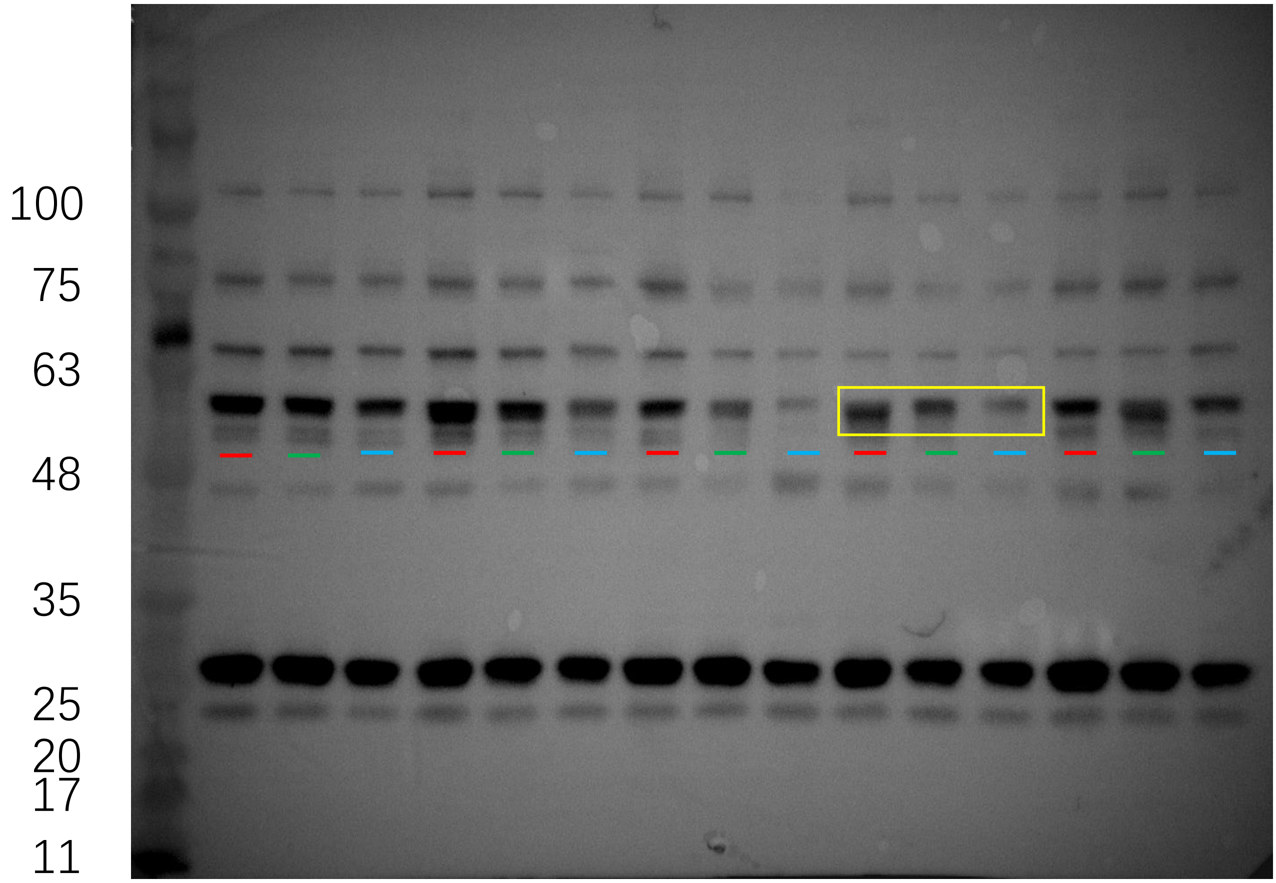

Fig.3D

GAPDH

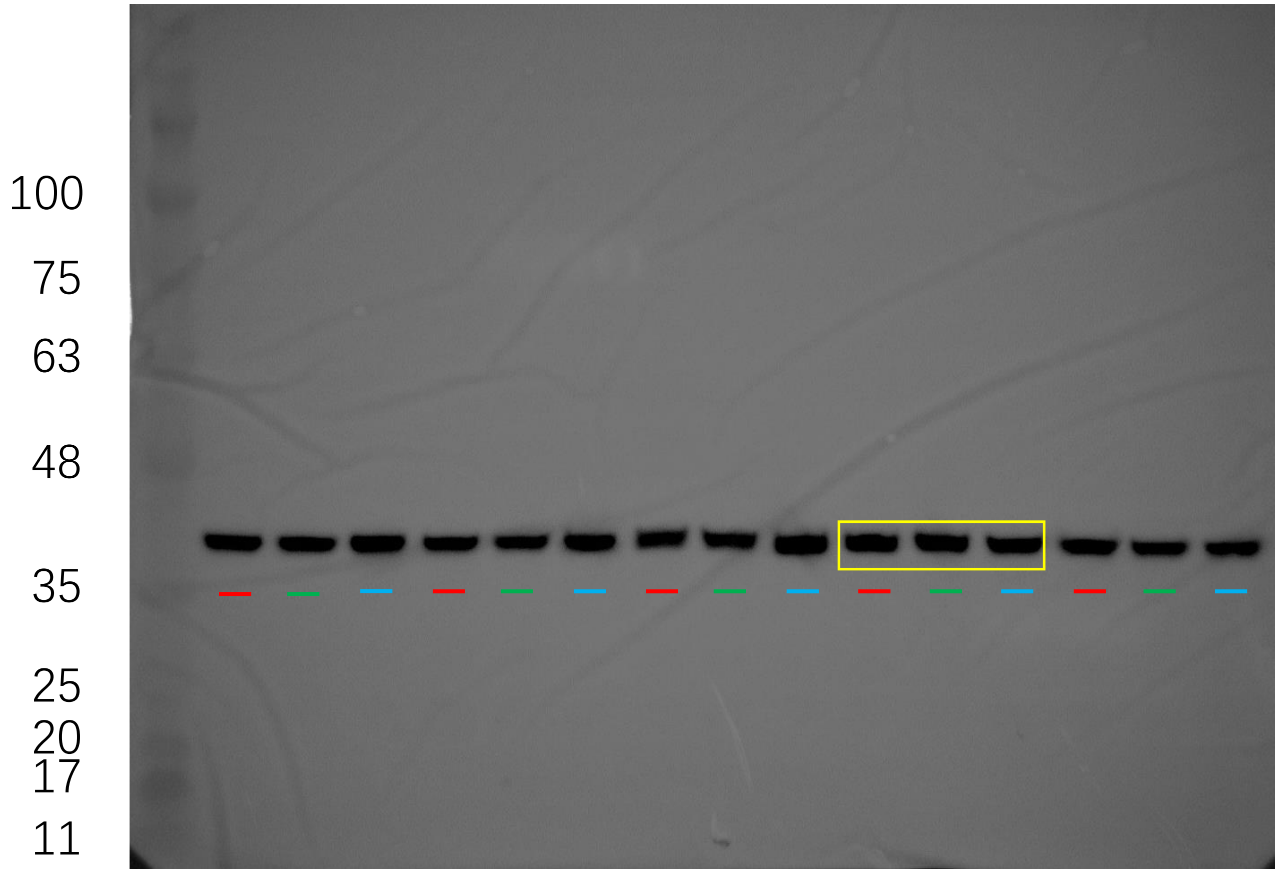

Fig.3D

MMP9

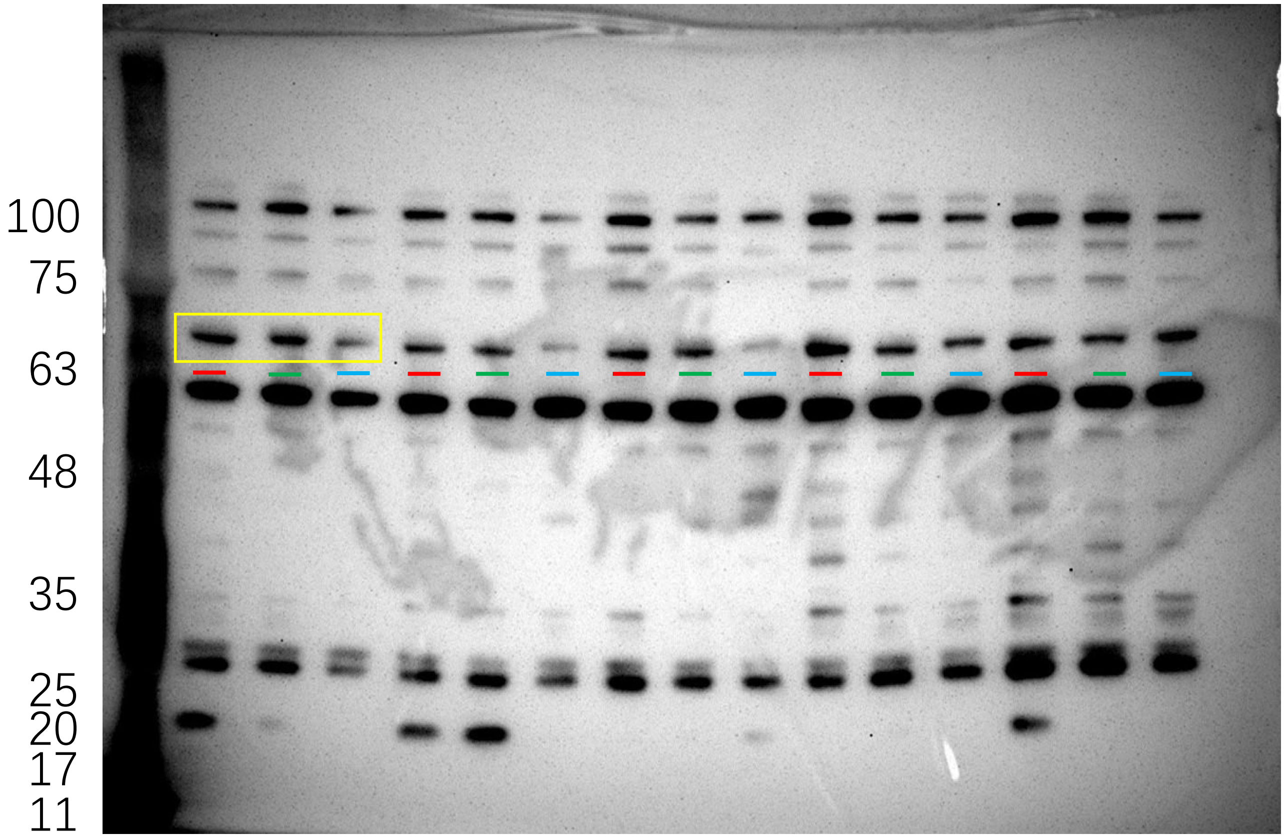

Fig.3E

GAPDH

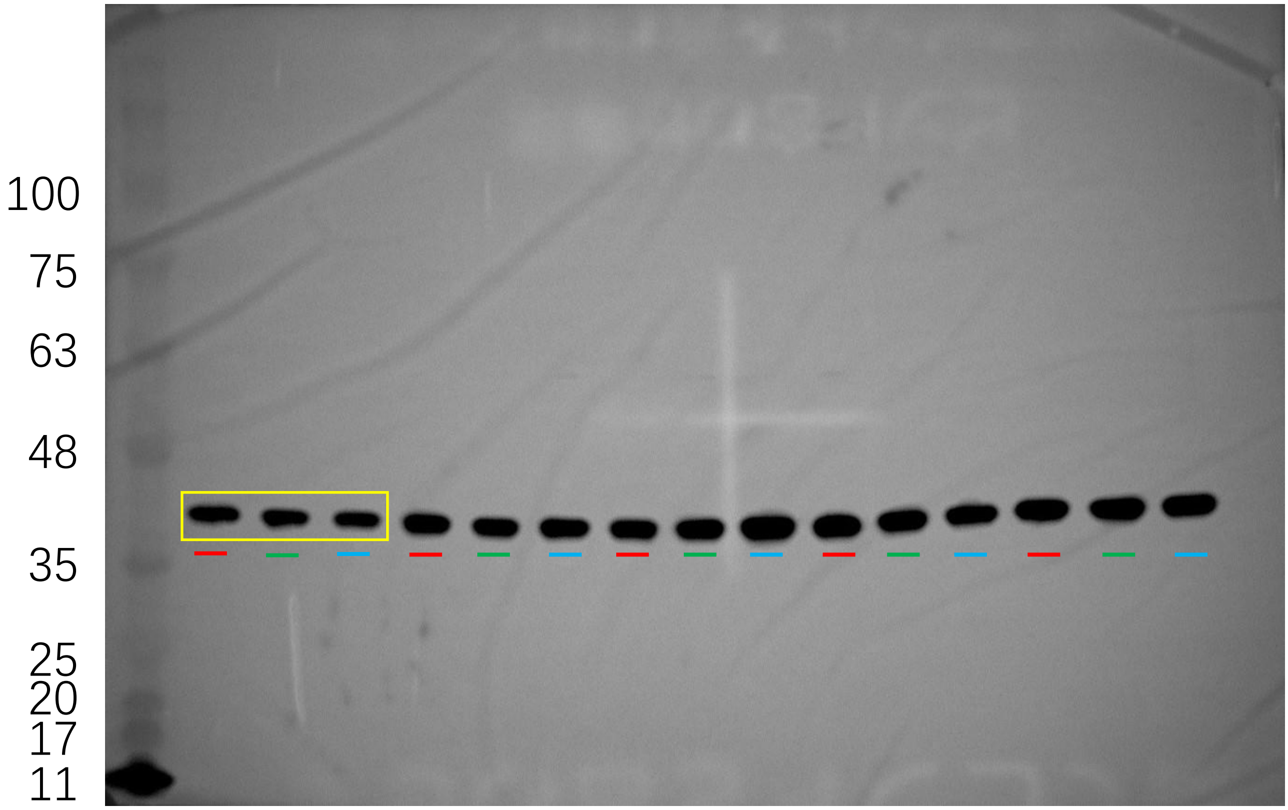

Fig.3E

iNOS

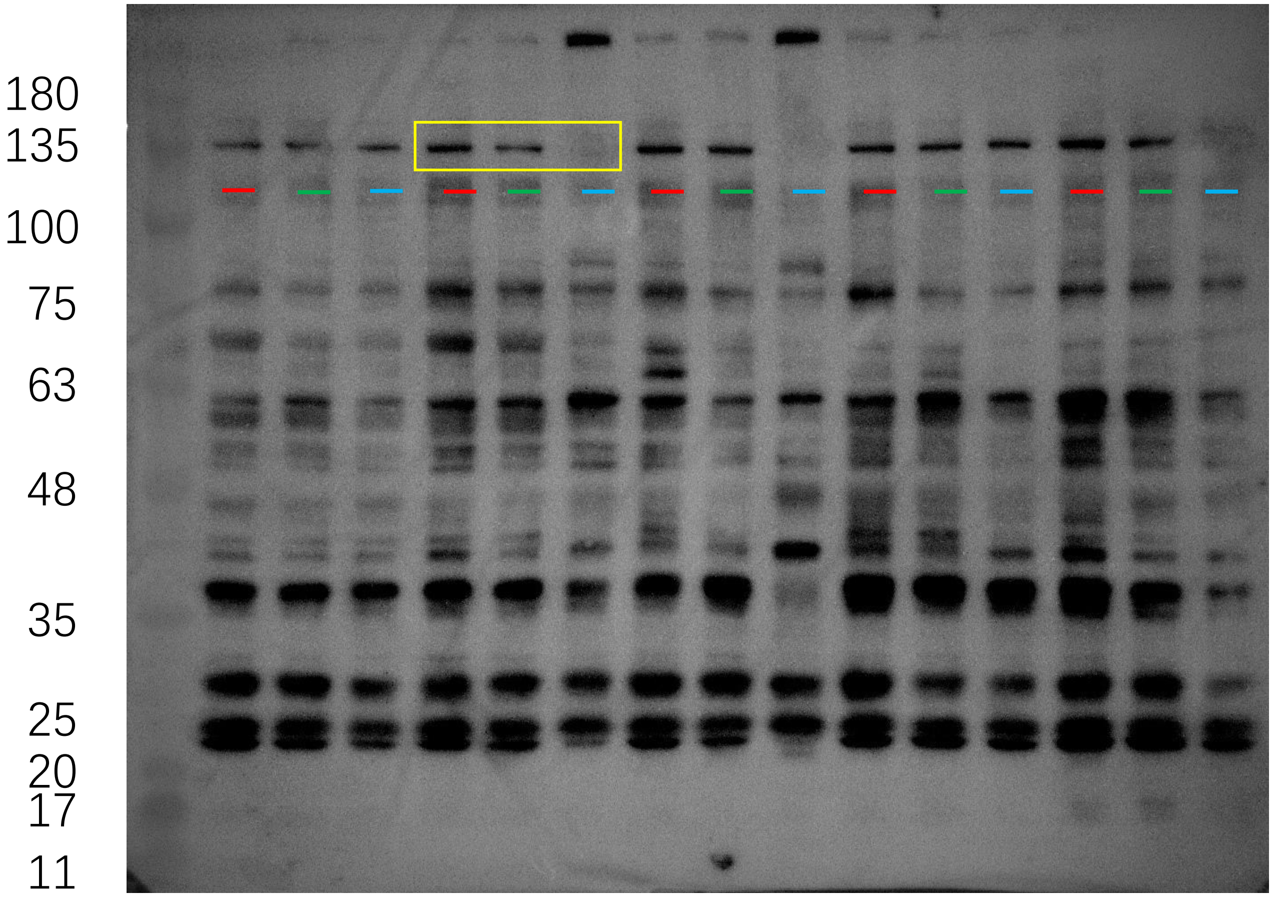

Fig.4A

GAPDH

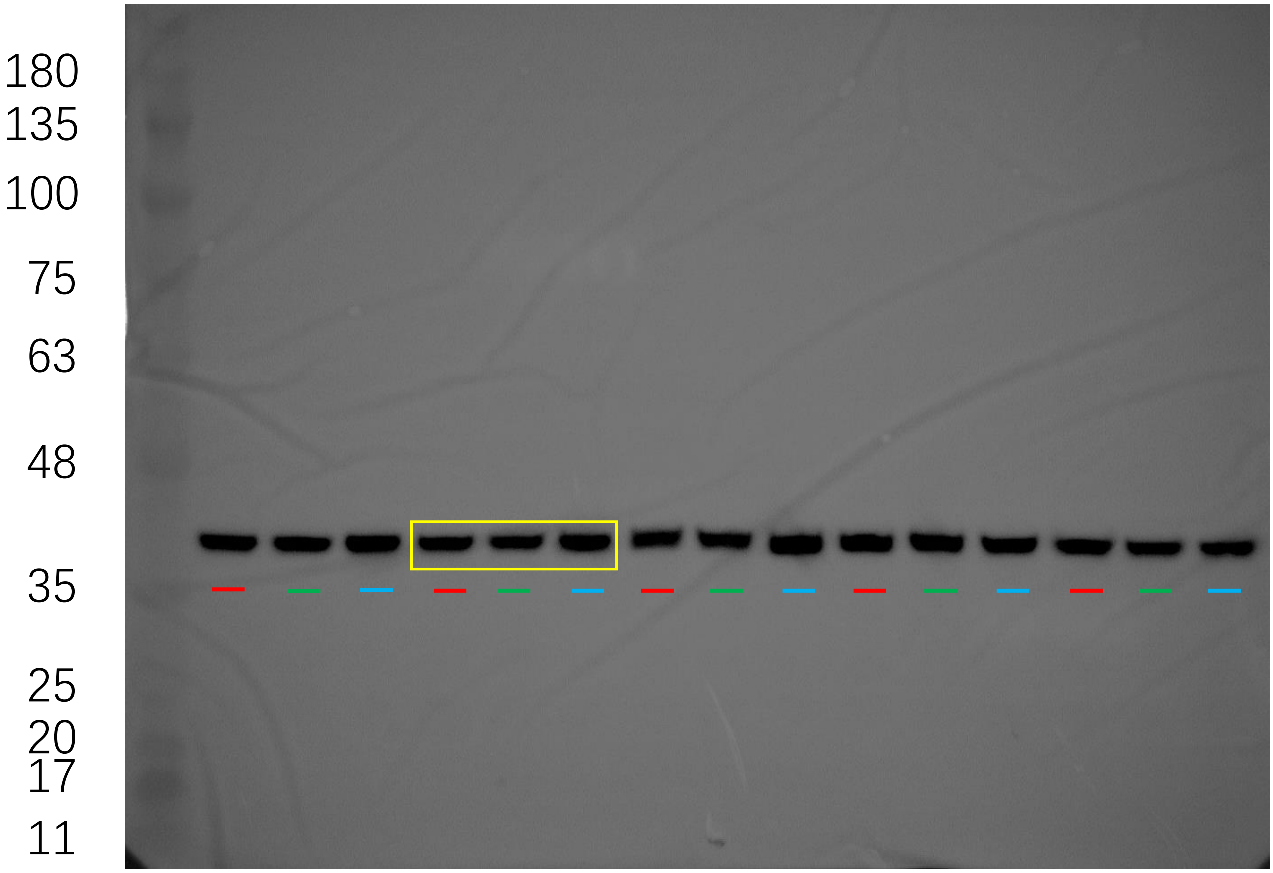

Fig.4A

HNE

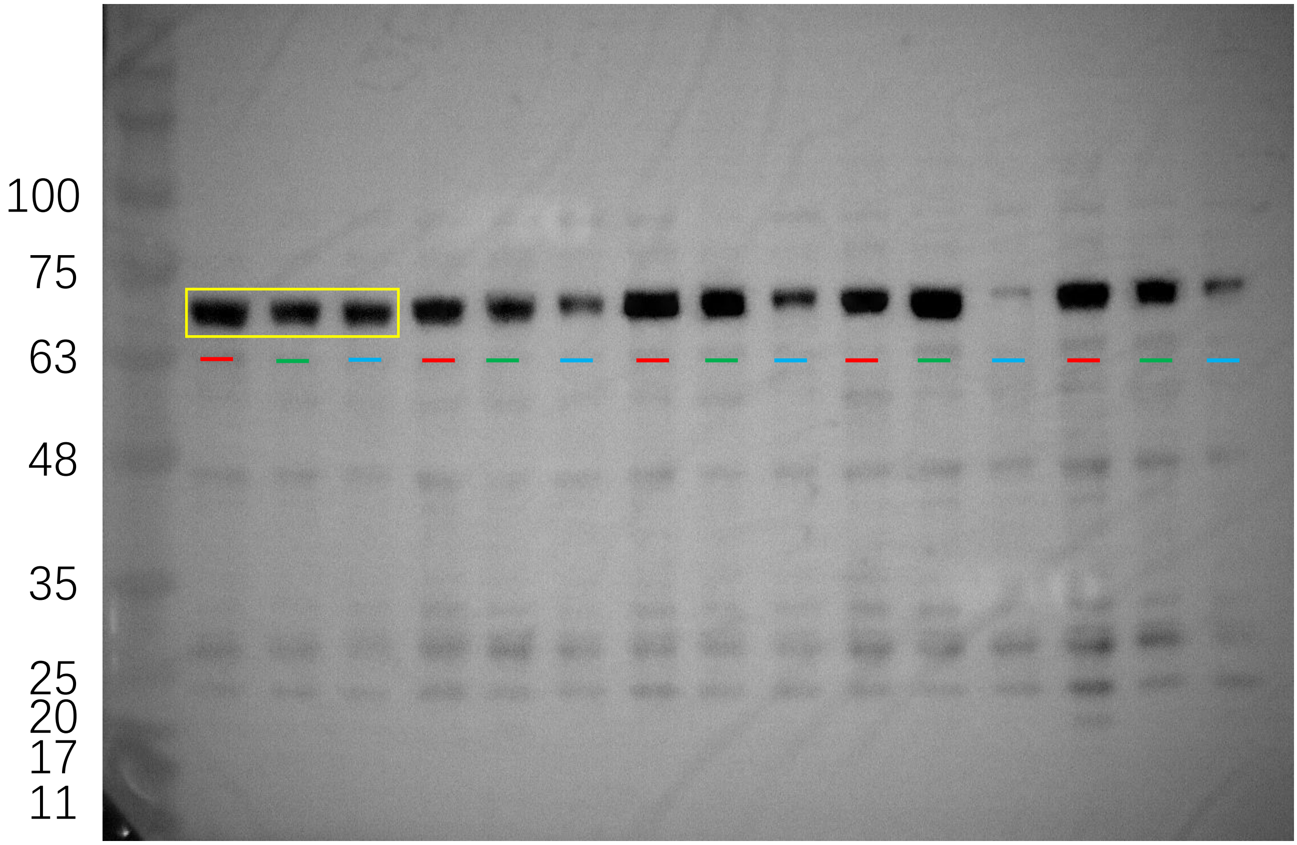

Fig.4B

GAPDH

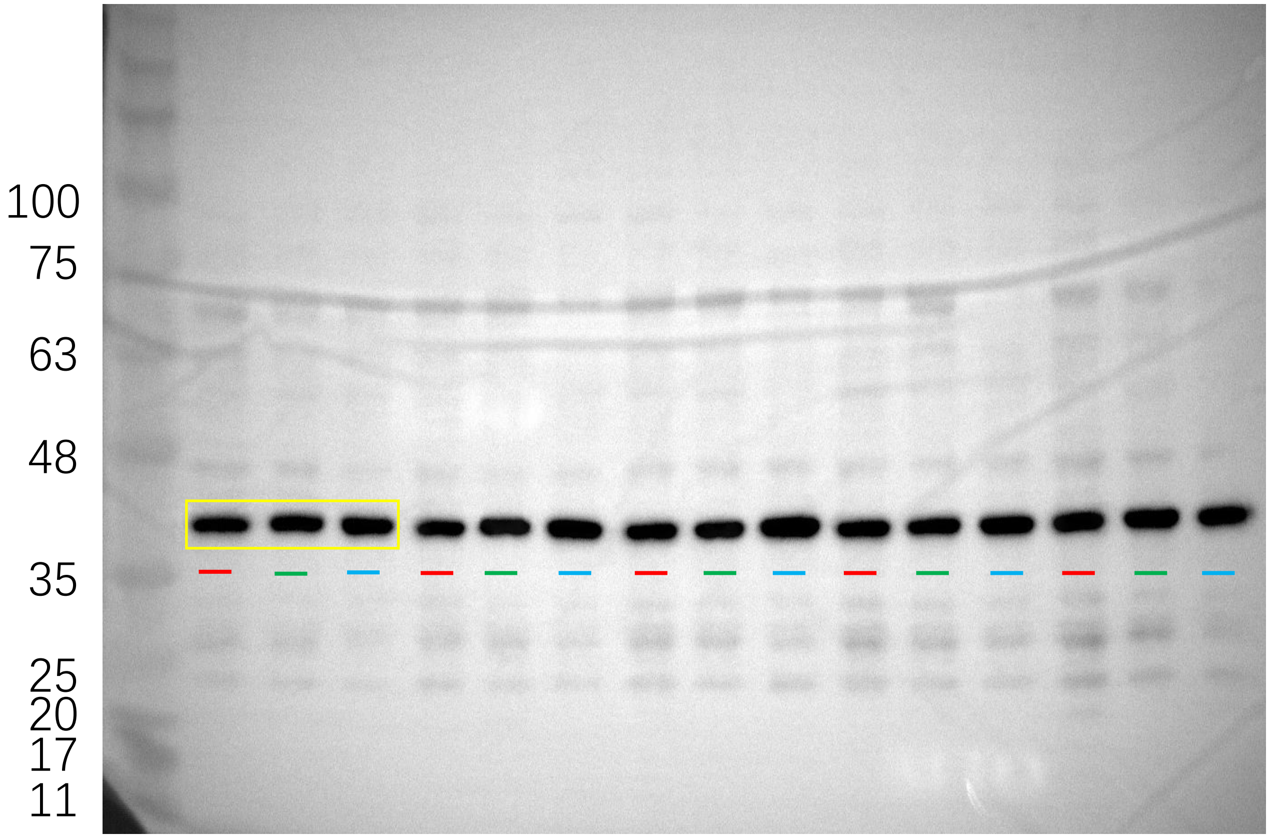

Fig.4B

SOD2

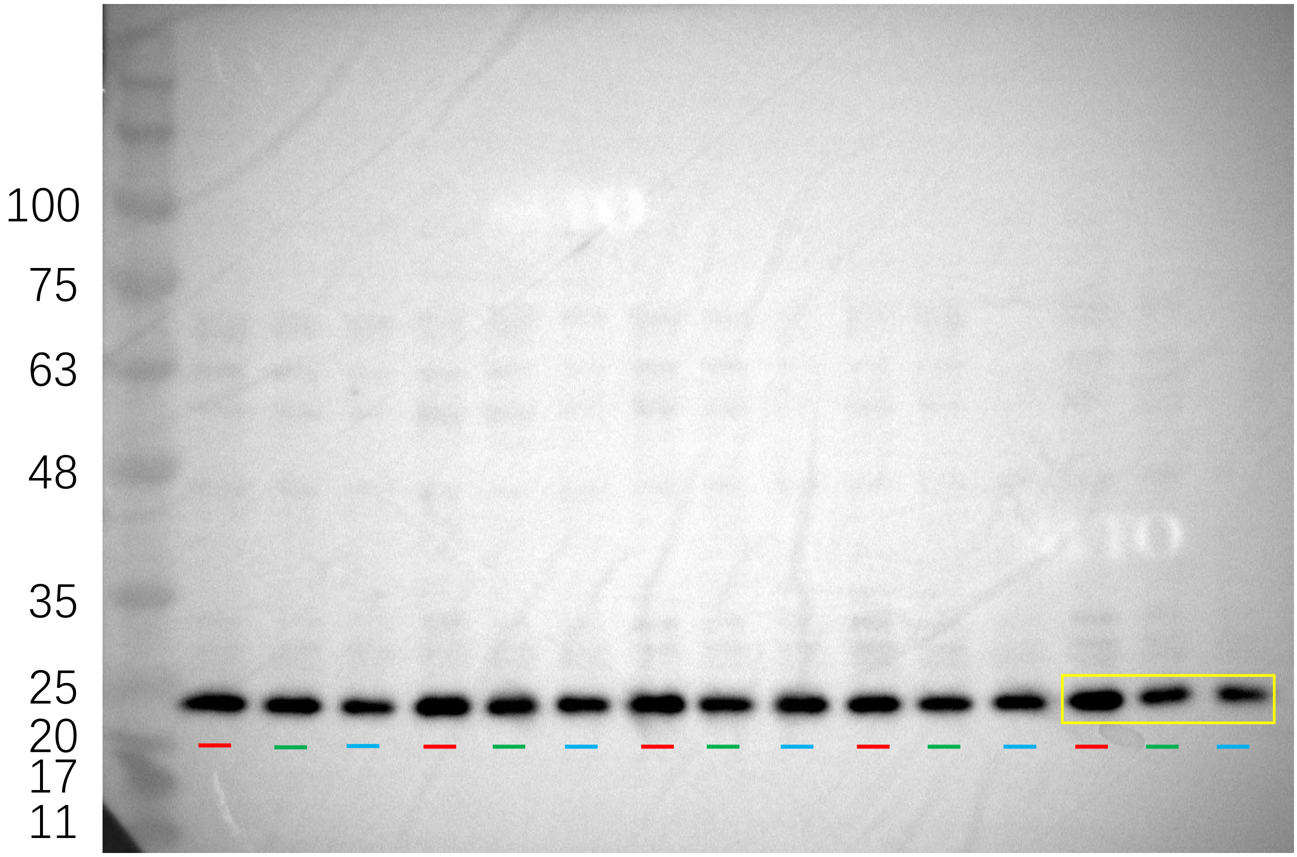

Fig.4C

GAPDH

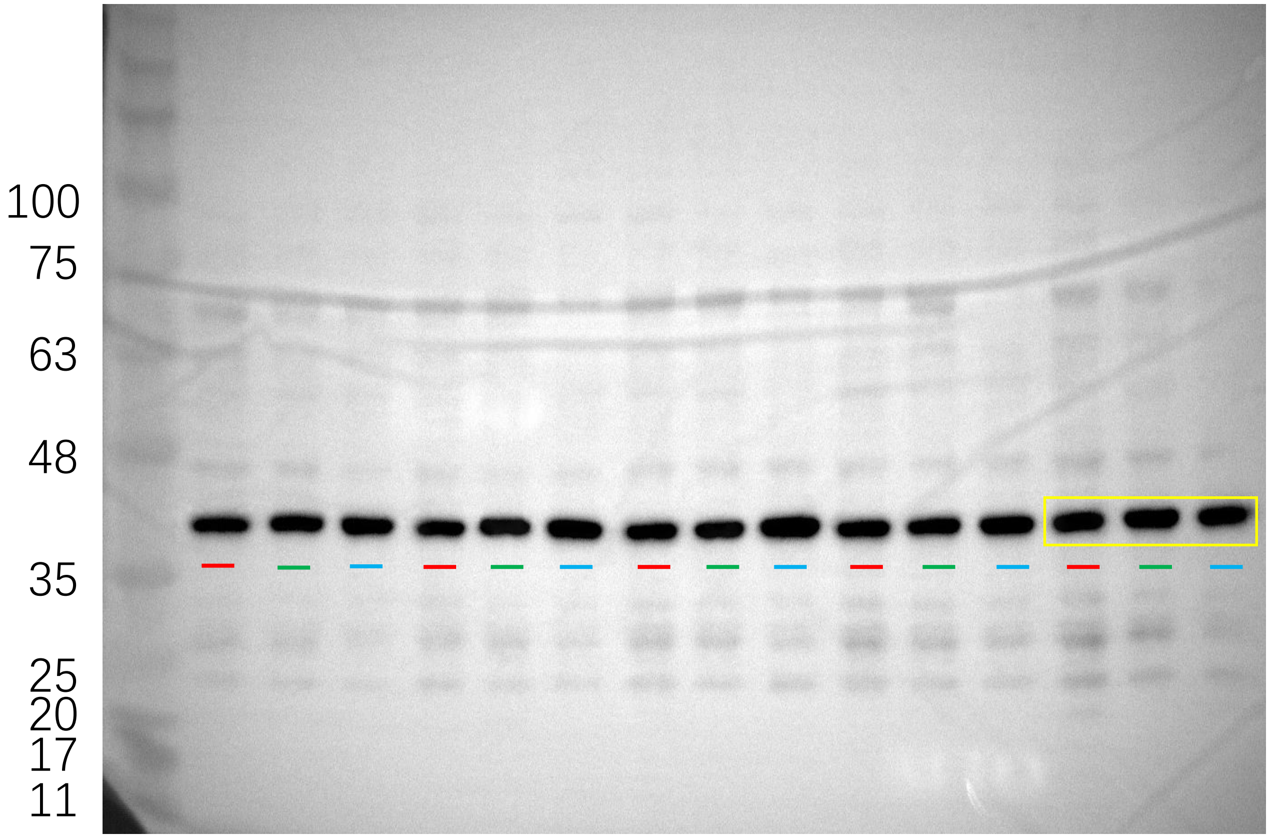

Fig.4C
