# Supplementary material for: Chewing Behavior Attenuates Lung-Metastasis-Promoting Effects of Chronic Stress in Breast-Cancer Lung-Metastasis Model Mice
Source: Cancers (Basel). 2022 Dec 1;14(23):5950. doi: 10.3390/cancers14235950 (PMC9740082; doi:10.3390/cancers14235950)
Supplement: Supplementary file 1 [file cancers-14-05950-s001.zip › cancers-1997202-supplementary/File S2.pdf]

File S2. The densitometry readings/intensity ratio of each band

— stress — stress+chewing — control

	1	2	3	4	5	6	7	8	9	10	11	12	13	14	15
GR/GAPDH	1.09	0.85	0.59	1.35	1.11	0.26	1.62	0.69	0.14	1.06	0.86	0.24	0.90	0.64	0.26
β2AR/GAPDH	0.68	0.63	0.54	1.07	0.69	0.76	1.18	0.97	0.30	1.06	0.84	0.71	0.91	0.88	0.60
TNF-α/GAPDH	0.93	0.60	0.20	1.11	0.71	0.16	1.30	0.68	0.07	0.99	0.24	0.34	0.81	0.78	0.68
TGF-β/GAPDH	1.02	0.88	0.71	0.98	0.89	0.45	0.86	0.61	0.31	0.79	0.90	0.75	1.06	0.86	0.76
VEGF/GAPDH	1.34	1.10	0.78	1.55	1.25	0.76	1.33	1.04	0.81	1.26	0.88	0.75	1.19	0.69	0.43
MMP2/GAPDH	1.31	0.89	0.78	1.26	0.84	0.49	0.77	0.42	0.14	0.63	0.34	0.20	0.91	0.65	0.42
MMP9/GAPDH	0.89	0.83	0.50	0.55	0.56	0.34	0.80	0.67	0.13	0.78	0.55	0.52	0.73	0.61	0.49
iNOS/GAPDH	0.82	0.67	0.51	0.91	0.42	0.13	0.99	0.67	0.11	0.70	0.62	0.56	1.09	0.67	0.10
HNE/GAPDH	1.22	0.93	0.90	1.14	0.91	0.43	1.50	1.05	0.42	1.01	1.19	0.23	1.06	0.80	0.69
SOD2/GAPDH	1.28	1.10	0.87	1.42	1.13	0.91	1.54	1.16	0.84	1.08	0.86	0.76	1.09	0.78	0.80
